# Of the Possible Cure of Suppurative Arthritis, with the Preservation of Mobility

**Published:** 1856-09

**Authors:** Hyppolite Blot

**Affiliations:** Chief of the Obstetric Clinic of the Faculty of Medicine of Paris


[From the Archives Generales, May, 1856.]

*Of the Possible Cure of Suppurative Arthritis with the Preservation of Mobility.* By Dr. Hyppolite Blot, Chief of the Obstetric Clinic of the Faculty of Medicine of Paris.

The object which I have proposed to myself to accomplish, is sufficiently indicated by the title of this essay. I wish to adduce facts to prove what I have not seen mentioned in any, either of our classical treatises, or in monographs on the diseases of articulations, viz.: that a termination of suppurative arthritis with the preservation of motion in the joint is, if not a common, yet at least a possible event.

To demonstrate the correctness of what I have stated in relation to the opinion of surgeons upon the different modes of termination of suppurative arthritis, it will be sufficient to adduce a few passages from the principal authors that I have been able to consult in relation to this subject.

Boyer and the surgeons who preceded him, do not describe at all the inflammations of the joints as distinct diseases; they include their history in that of white tumors, and, in relation to these, they agree in stating that, when these affections are complicated with purulent effusion into the joints, anchylosis is the most fortunate issue that can take place. To find these diseases separately considered, it is necessary to refer to treatises that are altogether modern.

In the *Dictionary of Medicine* we find Velpeau stating that "the least that can happen, when suppuration occurs in such cases, is an *irremediable anchylosis.* In other cases he may be so fortunate as to find the discharge to cease, at least in part; the general sympathies become quiet, the affection becomes purely local, permitting to the surgeon the possibility of a complete removal of the disease by amputation, or resection of the articulation."

In relation to articular ostitis, Sanson thus expresses himself: "Difficult to arrest, even in its incipient stage, it becomes almost impossible to check the disease when suppuration is established. We may then but very rarely hope for a cure, and that generally with an anchylosis of the bones."

In the same work, (El. de Path. Med. Chir., 4th Edit.,) in reference to traumatic arthritis, we read again: "When pus is formed in the interior of an articulation, the disease becomes much more serious. Imprisoned in a capsule, the pus effects a change in the synovial sac, the cartilages become eroded, and terminates by involving the spongy extremities in destructive caries. Sometimes a point of the articular capsule is destroyed, and the pus burrowing in the cellular tissue forms often extensive sinuses in which it accumulates and becomes decomposed. The life of the patient is then doubly compromised, by the abundant suppuration and by the effect of its resorption; *anchylosis is inevitable.*

M. Begin, in treating of traumatic arthritis, concludes his article relative to the prognosis and termination of the disease, as follows: "In the rarest and most fortunate cases, the secretion of pus gradually abates; from all the parts surrounding the joint, from the synovial membrane as well as the cartilages, cellular and vascular granulations arise, which, coalescing, obliterate the cavity of the joint and cause a firm and solid adhesion of all the contiguous parts. The joint of the patient is then *irremediably anchylosed.*"

In the same work, (Dict. de Med. et de Chir. Prat.,) in speaking of rheumatismal arthritis, M. Roche says: "In some cases the synovial becomes inflamed and suppurates, the cartilages become eroded and ulcerated, the bones become softened and carious, and there is no resource but in amputation or in resection of the joint."

M. Vidal is no less explicit. "In all cases in which it becomes necessary to make a prognosis," says this author, "it should be given with much caution and reserve, for it is either due to an internal cause when it is complicated and will recur, or it is of a traumatic origin, in which case it becomes extremely grave; for if the patient is cured, it will only be at the expense of the functions of the joint."

As to M. Nelaton, occupying a purely surgical point of view, and not having devoted a special article to the consideration of arthritis, he has not found it necessary to express an opinion upon the diverse modes of termination of this affection.

In the *Compendium of Surgery* the following occurs relative to acute arthritis: "When it terminates by suppuration and the formation of an abscess in the joint, we have everything to fear, and amputation may become necessary to save the life of the patient."

From all the citations that have been adduced, it is evident that authors are unanimous in the opinion that suppurative arthritis always presents an unhappy mode of termination, the patient sometimes succumbing from purulent infection, in others, amputation above the joint or resection becomes necessary; and again, in others, more rare and perhaps more fortunate anchylosis occurs, and the patient is cured with loss of motion of the articulation. I will only add that, having interrogated most of our masters of surgery in reference to their having observed any other mode of termination than those above indicated, I have uniformly received a negative response.

Besides, I have searched in vain for facts analogous to those which I shall report, in the rich collection of cases published by Brodie upon this subject as well as in most of the leading French periodicals.

It seems to me, therefore, interesting to report certain observations made already some time since, which conclusively establish the fact that another and more fortunate mode of termination of suppurative arthritis than those before indicated, is possible; to wit, *a cure with preservation of the motion of the joint.*

These observations, although but three in number in the human species, added to analogous ones related by our colleague, M. H., Bouley, at the Society of Biology, as occurring in the equine species, will suffice to prove the *possibility* of the mode of termination we have designated. Future researches will determine in what proportion of cases of suppurative arthritis we may venture to hope for so happy an issue.

One of these observations I owe to the kindness of M. Monod, who communicated to me the principal details of the case in 1848, during a conversation in reference to what I had myself seen. The second case was observed by myself in a patient introduced into the infirmary of the Maternity of Paris, to which I was then attached in the capacity of interne. Both cases occurred in females soon after parturition I will hereafter state what importance should be attached to this peculiarity. It is moreover to be well understood that we do not here treat of those multiple articular abscesses which are observed to occur in puerperal fever, but of mono-articular arthritis, freely developed and uncomplicated with any grave constitutional condition.

In regard to the third case, it is borrowed from the clinics of Professor Nelaton, who has kindly permitted me to treat of it in connection with the two preceding cases. This was a case of traumatic arthritis of the knee, developed in a young man of eighteen years.

Case 1. The female P., aged eighteen years, a laborer of good constitution and sanguine temperament, was born of healthy parents, and had herself never been sick. Menstruation commenced at sixteen years of age, and continued regularly until she became pregnant. During her entire pregnancy she suffered not the least illness.

The 20th Feb., 1848, without appreciable cause, she gave birth to a male child weighing 2500 grm.,* at the eighth month of her pregnancy. A vertex presentation in the ——. The birth was natural after a labor of eighteen hours. No accident occurred during the day, but during the night a violent attack of colic occurred for which she was conveyed to the Infirmary.

Feb. 21st. Simple cataplasms with laudanum were sufficient to calm the pains.

Feb. 23d. The abdominal pains had entirely ceased. She, however, complained of pain in the right foot, which she compared to spasmodic pain. No marked local affection, however, could be detected by the most careful examination. Slight redness and tumefaction on a level with the internal malleolus was all that was observed; but all movement of the tibio-tarsal articulation was very painful. Six leeches to the part tumefied; a bath followed with a large linseed cataplasm. To secure the influence of position, the limb was elevated upon a cushion, and to avoid the pain of motion, the foot was fixed by bandages to a hoop that sustained the covering. General condition good; no appreciable fever.

Feb. 24th. The patient was much relieved; she suffers now but very slightly, when the limb is moved. Poultice renewed twice a day.

Feb. 26th. The amelioration is not continued; the tumefaction has

* The gramme is about equal to 15 grains Troy.

increased, especially in the malleolar region; fluctuation, however, is not distinctly perceived. Treatment continued

March 3d. Since the 26th Feb. the tumefaction has continually augmented notwithstanding the means employed, and fluctuation in the malleolar region has become quite distinct. An incision, about an inch in length, was made on both sides of the joint, from which flowed a considerable quantity of laudable pus, mixed with strings of synovia, easily recognized by its yellowish color and syrupy consistence permitting it to be drawn out in long filaments. A soft probe introduced into the internal incision, penetrated more than two inches in depth and passed without difficulty into the tibio-tarsal articulation On withdrawing it, it was readily made to pass into several of the other tarsal joints by changing its direction. Without the joint the probe was arrested by fibrous bands. When the foot was moved upon the leg it caused a sharp pain, a rough friction sound, a sort of crepitation, could be distinctly heard and felt. This fact was confirmed by all the persons present at the time. Same position of the limb maintained, and the same treatment continued.

March 4th. Very evident improvement; pain of the joint less severe; suppuration more abundant; proportion of synovia greater than yesterday. Treatment continued.

March 9th. Suppuration diminishes from day to day, the pus becoming more liquid and the proportion of synovia increasing. Pains none when the foot is not moved Same treatment continued.

March 15th. Suppuration is completely arrested, and the pain has quite ceased. Even slight movements of the foot excite no pain. Same treatment continued.

March 18th. The incisions are nearly closed; the tumefaction having ceased, the joint is restored to its normal volume; the patient is able to move the foot without pain; the friction sound and crepitation have also ceased The cataplasm was replaced by a simple unguent.

March 26th. The incisions being healed, and the movements painless, the joint may be considered as completely cured.

March 29th. The articulation again and without assignable cause swollen and painful, and the surface somewhat red. Cataplasms. Elevation.

March 30th All the symptoms augmented, and apparently a slight fluctuation felt about the malleolous internus. As incision gives issue to nothing but blood. Treatment as before.

March 31st. Patient improved; suffers but slightly; incision uniting.

April 2d. Symptoms all disappeared. Treatment discontinued. Rest in bed.

April 5th to 17th. During this period the patient gradually acquired the power of using the limb without inconvenience, and left the maternity without the slightest trace of anchylosis or rigidity.

Case 2. Madame X., thirty-five years of age, of a nervous temperament and good constitution, had a very fortunate first accouchment with the exception of an unusual nervous prostration which lasted five or six hours. Lactation was quite normal.

She became pregnant a second time, and now suffered much more than during her former pregnancy. A removal and the cares of a large household caused her during the last months to undergo great fatigue. Her accouchment, however, took place at term without accident, but shortly after that, singular nervous phenomena again occurred, accompanied this time with anguish and insomnia. The mammary secretion was but slight, and towards the fourth or fifth day after accouchment, simultaneously with the cessation of the nervous symptoms, a serous effusion occurred in both knee joints which continued to increase notwithstanding every effort. At the end of a month, the effusion in the right knee was entirely absorbed, but the distension of the ligaments had been so great that the tibia was partially luxated outwards, and the motion of the articulation was considerably impaired.

In the left knee resorption of the fluid could not be obtained, and the joint remained greatly distended. Perfect rest with cauterization were quite ineffectual. During the second month, an acute inflammation supervened without appreciable cause, terminating in suppuration; a spontaneous and abundant discharge of pus soon occurred from the inferior and external part of the articulation. No serious constitutional symptoms occurred; several counter-openings were made at different points, and the healthy suppuration gradually diminished. About a month after the opening of the purulent abscess of the joint, the knee was cured. From that moment, the motion of the joint was gradually restored, but the power of flexion could never be carried beyond a right angle.

CASE 3 *Traumatic suppurative arthritis cured without loss of motion of the joint* A shawl maker, eighteen years of age, punctured the left knee joint with the point of the scissors employed for shearing the shawls. At first, he gave very little attention to the injury, but after the eighth and tenth day, a considerable swelling of the joint occured, and the patient solicited and obtained permission to enter the Hospital St. Louis.

The case presented all the signs of a penetrating wound of the knee. A very extensive, white, œdematous tumefaction had taken place resembling that of *phlegmasia alba dolens*. The lips of the wound were flabby, whitish and œdematous. A sero-purulent liquid issued from the wound, increased by pressure upon different points of the articulation. Adopting the treatment extolled by M. Fleury, the entire joint was enveloped in a vesicating plaster.

Notwithstanding this measure, the tumefaction remained unabated. The liquid flowing from the wound became more and more purulent, until it no longer contained any serum, and every day a considerable discharge from the original wound and from the counter-openings took place. Perfect rest in a wadded splint.

Somewhat later, the discharge again became more serous, which character augmented until it finally ceased. In six weeks, the wound was completely closed. The power of motion in a slight degree still existed. The difficulty at first existing became less and less, and at the end of three months, the patient presented himself to M. Nelaton, having completely recovered the motion of the joint. He now returned to his former occupation of shawl-making.

To the preceding cases I will add another, addressed to the Society of Biology, by Prof. H. Bouley, of Alfort. Our colleague presented the temporo-maxillary articulation of a horse, in which suppurative arthritis had existed for some time. A considerable quantity of pus flowed from the diseased joint, especially during mastication to which the animal was urged, notwithstanding the pain it produced, by the intensity of hunger. Desiring to ascertain the state of articulation, M. Bouley sacrificed the animal, and on examination found that the cartilages from both the temporal and the inferior maxillary surfaces had completely disappeared, and were replaced by red and vascular vegetations, densely crowded together, covered in spots with smooth osseous plates. The synovian membrane had entirely disappeared.

M. Bouley is of the opinion that the joint which, at the death of the animal, was suppurating but little, was in process of healing, and founds this op nion upon observations in a considerable number of other cases in the same species of animal. It appears, indeed, that suppurative arthritis of the temporo-maxillary articulation, is not rare in the horse, and it is often observed that the animal, urged by hunger, maintains the freedom of the joint by use, and a complete cure is effected after a longer or shorter period.

It is scarcely to be expected that similar observations should be made upon other joints, especially upon those of the legs; for, as is well known, when a horse has received so serious an injury as to be incapable of any service, he is not deemed worthy of preservation.

Such are the facts to which I desire to call the attention of surgeons.

I will endeavor now in a few words to present the reflections that flow very naturally from them.

When we seek to account for the happy exceptions which I have presented, two interpretations present themselves prominently to the mind, one having reference to the special conditions in which the first two patients were placed; the other may be applied to the mode of treatment which one of them received. The two cases referred to, presented themselves in fact during the puerperal period. May not this condition itself furnish or suggest an explanation of the cause of the fortunate issue of these cases? I am inclined to adopt that opinion.

The facility, and especially the rapidity with which pus is formed in the puerperal state, may furnish an explanation of the effusion and accumulation of pus in joints, without the occurrence of such profound lesions of the synovial membranes and cartilages as to render anchylosis inevitable; the transformations have not had time to become so grave as to prevent the articular surfaces from returning to their normal condition and a consequent restoration of their function of motion. This view of the subject, yet hypothetic, may hereafter acquire the value of a demonstration, if opportunities occur of examining the elements of the joints affected with suppurative arthritis in puerperal females, who have succumbed from intercurrent diseases.

But until direct proof can be adduced, the hypothesis here put forth may apparently derive support from the peculiar facts observed in our second case, to-wit, the presence of a distinct quantity of pure synovia mingled with the laudable pus furnished by the joint; the relative quantity of the former always increasing as the arthritis progressed towards a cure. How, indeed, can the continued secretion of synovia be explained without admitting that the synovial membrane has preserved its integrity, at least to some extent? This observation seems moreover to possess some direct practical utility. From the light of this observation, the surgeon should, if I am not mistaken, be capable of making a clearer diagnosis in a given case. The prognosis being as much less serious, and the prospect of cure the greater as the proportion of synovia in the fluid that issues from the joint when opened, is augmented. This hopeful anticipation being moreover enhanced, if, while from day to day the synovia increases, the purulent secretion suffers a corresponding diminution.

The preceding reflections are especially applicable to the first two cases, and might cast a doubt upon the probability of obtaining similar success in ordinary cases. But the third case is quite different. In this, we find a genuine suppurative arthritis not occurring in a puerperal female, but in a young man of eighteen years. Taken in connection with the case of comparative pathology above cited, this case seems to compel the admission that suppurative arthritis is susceptible of cure without loss of motion of the articulation.

As to the treatment, perhaps, that was not entirely foreign to the success obtained. It will be recollected that, as soon as suppuration became manifest, free incisions were made on both sides of the joint.

By thus avoiding the serious accidents indicated by all writers on this subject, as the separation and infiltration of muscles, denudation of bones, &c., &c., perhaps, also by thus diminishing the granulations of the synovial membrane, the wasting of the cartilages, the inflammation, suppuration, a d sometimes the necrosis of the osseous extremities of the articulation, we shall augment the chances of curing the diseased joints and preserving, if not the complete, yet the greater part of the mobility of the articulation.

I am well aware that, at first sight, these views seem to be contradicted by what daily experience teaches in regard to the gravity of wounds communicating with joints; indeed, it is well known that such lesions are the more seirous, and more frequently followed by suppuration in proportion to the size of the wound and the greater facility for entrance of air into the cavity of the articulation. These objections, however, are believed to be more specious than well founded, for the two elements of the comparison are not analogous. In one case, we dread to see suppuration supervene, in the other it has already occurred, and measures are not indicated to avoid it, but to render it as harmless as possible. The best means for attaining this object will probably be to avoid the prolonged contact of the pus with the synovial surfaces, by giving it a free and early exit.

It is hardly necessary to add that these reflections are submitted to the mature judgment of our distinguished surgeons with great reserve and hesitation.

Whatever may be the influence of treatment, the cases of which we have given the principal details prove that in suppurative arthritis the surgeon ought not to despair of curing the patient, and preserving the functional integrity of the limb.—*Peninsular Jonrnal.* A. S.

